# Hydrophobic profiles of the tail anchors in SLMAP dictate subcellular targeting

**DOI:** 10.1186/1471-2121-10-48

**Published:** 2009-06-19

**Authors:** Joseph T Byers, Rosa M Guzzo, Maysoon Salih, Balwant S Tuana

**Affiliations:** 1 Department of Cellular and Molecular Medicine, 451 Smyth Road, University of Ottawa, Ottawa, Ontario K1H 8M5, Canada

## Abstract

**Background:**

Tail anchored (TA) membrane proteins target subcellular structures via a C-terminal transmembrane domain and serve prominent roles in membrane fusion and vesicle transport. Sarcolemmal Membrane Associated Protein (SLMAP) possesses two alternatively spliced tail anchors (TA1 or TA2) but their specificity of subcellular targeting remains unknown.

**Results:**

TA1 or TA2 can direct SLMAP to reticular structures including the endoplasmic reticulum (ER), whilst TA2 directs SLMAP additionally to the mitochondria. Despite the general structural similarity of SLMAP to other vesicle trafficking proteins, we found no evidence for its localization with the vesicle transport machinery or a role in vesicle transport. The predicted transmembrane region of TA2 is flanked on either side by a positively charged amino acid and is itself less hydrophobic than the transmembrane helix present in TA1. Substitution of the positively charged amino acids, in the regions flanking the transmembrane helix of TA2, with leucine did not alter its subcellular targeting. The targeting of SLMAP to the mitochondria was dependent on the hydrophobic nature of TA2 since targeting of SLMAP-TA2 was prevented by the substitution of leucine (L) for moderately hydrophobic amino acid residues within the transmembrane region. The SLMAP-TA2-4L mutant had a hydrophobic profile that was comparable to that of SLMAP-TA1 and had identical targeting properties to SLMAP-TA1.

**Conclusion:**

Thus the overall hydrophobicity of the two alternatively spliced TAs in SLMAP determines its subcellular targeting and TA2 predominantly directs SLMAP to the mitochondira where it may serve roles in the function of this organelle.

## Background

The tail-anchored (TA) membrane proteins include diverse family members such as cytochrome b5, DMPK A, & C, Bcl-2, Tom, and Sec61 β & γ which are critical for cell function [1-8]. Several of these proteins, such as Synaptobrevin, are important in vesicle transport and membrane fusion [9]. The tail-anchor of TA proteins is defined as the C-terminal hydrophobic transmembrane domain which may be flanked by hydrophilic amino acid residues [9,10]. The Tail-Anchors can target proteins to a wide range of subcellular compartments including the ER (endoplasmic reticulum) [11-13], the MOM (mitochondrial outer membrane) [14-17], peroxisomes [18,19], the perinuclear membrane [20] and the chloroplast outer envelope in plants [21]. The molecular mechanism by which tail anchored proteins target specific membranes is of much interest. There appears to be no consensus amino acid sequence in the different TAs of proteins that dictate their targeting. In fact the sequence of the amino acids in the transmembrane domain has been shown to be irrelevant in synaptobrevin, where a poly-leucine tail (13 amino acids) targets the protein to the ER [22]. However, all tail anchors are predominantly hydrophobic in nature, as determined from the amino acid sequence of the membrane spanning region. Some studies indicate that there may be recognition motifs contained within the TA for proteins such as PEX26 and PEX15p, which cause them to target the peroxisome [23]. No such motifs have been identified for TAs which target proteins to the ER or the MOM. However, some TAs which target proteins to the MOM, are flanked by positively-charged residues adjacent to the membrane-spanning region which are believed to dictate their targeting to either the ER, or both the ER and the MOM [2,24-26]. This has led to the view that the p ositive charges individually or collectively in and around the TA determines its subcellular targeting [21,26]. Other studies by Biellharz *et al*., [20], Brambillasca *et al*., [27] and Wattenberg *et al*., [28] have provided evidence that the hydrophobicity of a tail anchor sequence itself can also influence which organelle it will target. Weather this is a unifying concept involved in targeting all TAs remains to be explored. The Sarcolemmal Membrane Associated Protein (SLMAP, formerly known as SLAP) is a tail-anchored protein which can carry two alternatively spliced TAs [8,29]. All SLMAP isoforms are encoded by one gene and many SLMAP isoforms have now been shown to be expressed in a tissue specific manner with proposed roles in myoblast fusion, excitation-contraction coupling and centrosomal organization [8,29-32]. The largest SLMAP isoform, the 91 kDa SLMAP3, is ubiquitously expressed in all tissues whilst the smallest isoform, the 34 kDa SLMAP1, is only expressed in cardiac and muscle tissue [30]. Each SLMAP isoform shares the C-terminal region of the protein but the smaller isoforms do not possess the N-terminal structures such as the FHA domain [8].

We have previously shown that when TA1 or TA2 are encoded as part of SLMAP1 they direct it to subcellular membrane structures [31] but which organelles they target and how, has not been defined. In addition, we have found that SLMAP carrying either TA1 or TA2 can be differentially expressed in the same tissue [8] Further more, immunohistological and biochemical analysis implies that SLMAP localizes in different subcellular compartments within the cardiomyocytes including the sarcolemma, SR/ER, and the transverse tubules [29,31]. TA1 comprises 27 amino acid residues with a predicted transmembrane helix of 18 residues and TA2 comprises 30 amino acid residues with a predicted transmembrane helix of 19 residues [8]. In this study we identify how TA1 and TA2 affect the subcellular targeting of SLMAP1 in Cos7 cells and provide evidence which supports the view that the overall hydrophobic profile of a tail anchor is critical for determining its subcellular localization. Further more, our analysis indicates that SLMAP1 itself is not involved in vesicle transport but can target the ER as well as the mitochondria, possibly the MOM, where it may play previously unrecognized roles in the subcellular function of these organelles.

## Results

### SLMAP is a component of intracellular membranes in non-muscle cells

**Figure 1:**
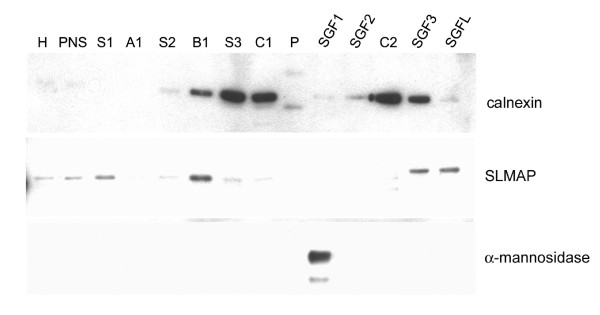
**Sub cellular distribution of SLMAP from liver**. Rat liver homogenate was fractionated by sucrose gradient centrifugation. Western blot analysis demonstrated that the cis-Golgi marker, α-mannosidase resides in the stacked Golgi fraction 1 (lane SGF1). Endogenous SLMAP (91 kDa) proteins are distributed among fractions where the ER marker calnexin is expressed (lanes B1; S3; SGF3). H (homogenate); PNS (postnuclear supernatant); SGF (stacked Golgi fraction); P (pellet).

The SR is derived from smooth endoplasmic reticulum, an extensive membrane-bound organelle, which contributes to general intracellular calcium regulation in eukaryotic cells [33]. In view of the abundant SLMAP expression in the SR of cardiac cells and the ubiquitous distribution of the 91 kDa SLMAP varient [30], we sought to determine whether this SLMAP isoform is also a component of membrane systems (ER, Golgi) in non-muscle cells. Microsomes were prepared from the postnuclear supernatant of rat liver homogenates and further separated into ER and Golgi fractions by sucrose gradient centrifugation (Figure 1) [34]. Protein fractions from each gradient were collected and separated by SDS-PAGE and subjected to immunoblot analysis using anti-SLMAP antibodies as well as specific markers of the ER (calnexin) and a cis-Golgi marker (α-mannosidase). A 91 kDa SLMAP isoform, consistent with the molecular size of the full length SLMAP protein, distributed with calnexin in fractions B1 and SGF3 but was not enriched in the fractions with the highest levels of calnexin (S3, C1 and C2) (Figure 1). SLMAP was absent in the stack Golgi fraction where α-mannosidase was enriched, and was present in SGF3 and SGFL together with some calnexin (Figure 1). These data suggest that SLMAP is not a resident of the Golgi although it may be present in specific regions of the ER in the liver. It seems likely that the difference between the distribution of SLMAP and calnexin is due to a combination of the properties of the tail anchors in the liver SLMAP as well as the lipid composition in the different forms and regions of the ER.

### Tail anchors in SLMAP target distinct subcellular membranes

**Figure 2:**
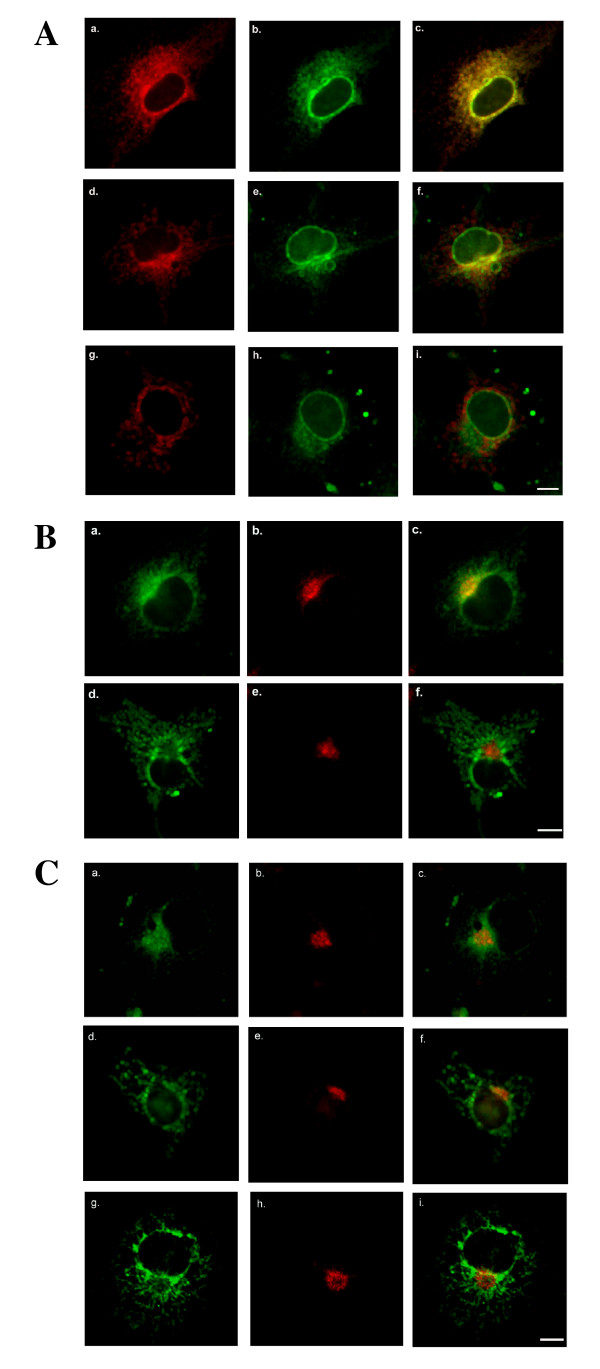
**Subcellular membrane distribution of SLMAP**. (A) COS7 cells transiently transfected with 6Myc-tagged SLMAP variants encompassing either TA1 (A; a-c), TA2 (A; d-f) or ΔLZ_TA2 (g-i) were stained with anti-calnexin polyclonal antibodies (b, e, h). The overlay of the anti-Myc and calnexin in these cells is shown in A; c, f, i. SLMAP distribution with respect to the secretory system. (B) COS7 cells transfected with either 6Myc-SLMAP-TA1 (B; a-c) or 6Myc-SLMAP1-TA2 (d-f) were stained with anti-SLMAP (B; a, d) and anti-ERGIC-53 (B; b, e), with the overlay shown in B; c, f. SLMAP distribution with respect to the Golgi apparatus. (C) COS7 cells expressing either 6Myc-SLMAP-TA1 (C; a-c); 6Myc-SLMAP-TA2 (C; d-f) or 6Myc-SLMAPΔLZ-TA2 (g-i), were co-stained with anti-Golgi 58 K (C; b, e, h) and anti-SLMAP (a, d, g), with the overlay shown in C; c, f, i. Scale bar = 10 μm.

In order to determine weather the two carboxyl-terminal TAs utilized by SLMAP can target to distinct membrane structures, 6Myc-tagged SLMAP1 carrying TA1 or TA2 was transfected in COS7 and its localization monitored relative to various components of intracellular organelles. SLMAP1 is a 34 kDa isoform which is expressed predominantly in cardiac and muscle tissue, and was found to be an integral membrane protein [30]. Co-staining with the ER marker calnexin demonstrated significant co-distribution with 6Myc-SLMAP1-TA1 at reticular formations (Figure 2A; a–c); however limited co-distribution was observed in cells expressing 6Myc-SLMAP1-TA2 (Figure 2A; d–f). Since SLMAP contains a coiled-coil leucine zipper involved in homodimerization [32], we investigated whether this motif will influence targeting. As shown, (Figure 2A; g–i) a deletion mutant lacking the leucine zipper (ΔLZ) 6Myc-SLMAP1-TA2ΔLZ had identical targeting when compared to the undeleted, expressed protein. Anti-ERGIC-53 monoclonal antibodies were used to detect the ER-Golgi intermediate compartment and consistently labelled fine punctuate structures concentrated at perinuclear sites in COS7 cells [35,36]. This distribution pattern was distinct from that of either 6Myc-SLMAP1-TA1 (Figure 2B; a–c) or 6 Myc-SLMAP1-TA2 (Figure 2B; d–f). Whereas Myc staining demonstrated that TA1 and TA2 direct SLMAP1 to perinuclear sites, no co-localization with the anti-Golgi58K monoclonal antibody was noted (Figure 2C; a–i). Further, the fungal metabolite Brefeldin A, a widely used agent that disrupts the structure of the Golgi apparatus [37,38] did not alter the subcellular distribution of SLMAP1 (data not shown).

### Disruption of the cytoskeleton and SLMAP localization

**Figure 3:**
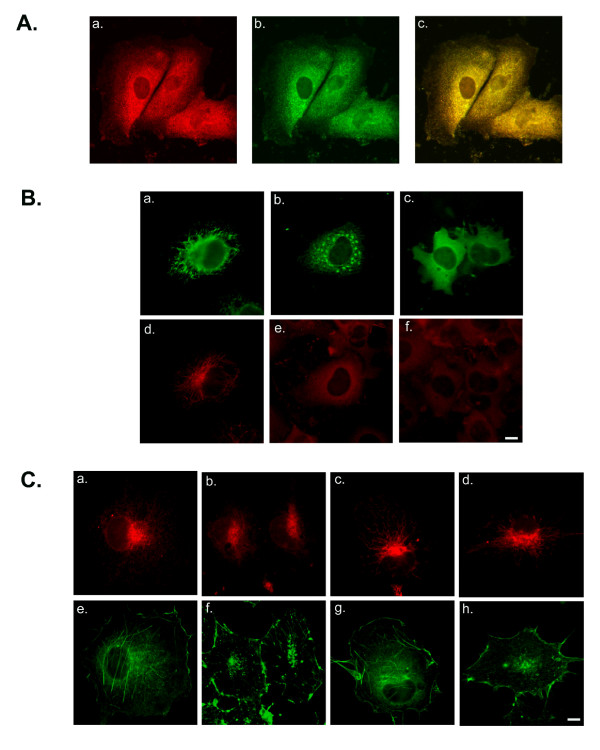
**SLMAP localizes to the ER and its associations are microtubule dependent**. (A), COS7 cells were stained with anti-SLMAP (A; a) and anti-calnexin (A; b) and merged image (A; c). (B), COS7 cells were transfected with 6Myc-SLMAP-TA1 (B; a, b, d, e) and 6Myc-SLMAPΔTM (B; c, f) and stained with Anti-SLMAP (B; a-c) and anti-α-tubulin (B; d-f). Cells treated with nocodazole (B; b, c, e, f). (C) COS7 cells transfected with either 6Myc-SLMAP-TA1 (C; a, b, e, f) or 6Myc-SLMAP-TA2 (C; c, d, g, h) and stained with phalloidin (C; e-h) and anti-Myc (a-d). Treatment with cytochalasin D (C; f, h) did not alter the distribution of the 6Myc-SLMAP-TA1 (C; b) or 6Myc-SLMAP-TA2 (C; d). Scale bar = 10 μm.

We examined the localization of endogenous SLMAP with anti-SLMAP antibodies and compared this with the ER marker calnexin using anti-calnexin antibodies and a clear co-distribution of these two proteins is evident (Figure 3A; a–c). The importance of cytoskeleton for the organization of the membrane organelles such as the ER has been demonstrated by the pharmacological disruption of microtubules [33]. In cells treated with the microtubule depolymerising agent nocodazole, the ER membrane collapses and aggregates around the nucleus [39-41]. Under conditions that disrupt microtubules, the distribution of the ectopically expressed ER-membrane associated SLMAP1 variant (6Myc-SLMAP1-TA1) was altered from a reticular-like distribution (Figure 3B; a) to an aggregate-like distribution (Figure 3B; b). These nocodazole-induced structures were not however observed in COS7 cells expressing SLMAP1 deletion mutant lacking the TA (6Myc-SLMAP1ΔTA) (Figure 3B; c). The effect of nocodazole on microtubule assembly was confirmed by staining with α-tubulin, which illustrated a distinct redistribution of the cytoplasmic microtubules (Figure 3B; d–f). These observations imply that SLMAP1-membrane associations require an intact microtubule network and would be consistent with the distribution of SLMAP1-TA1 at the ER membrane.

To determine whether SLMAPs are associated with the actin cytoskeleton, COS7 cells expressing 6Myc-SLMAP1 fusion proteins were treated with cytochalasin D, an actin myofilament disrupting agent [42]. Depolymerization of the actin filaments with cytochalasin D did not alter the localization of 6Myc-SLMAP1 fusion proteins carrying either TA1 (Figure 3C; a, b) or TA2 (Figure 3C; c, d), whilst inducing actin-containing microfilaments to change from filamentous (Figure 3C; e, g) to punctuate structures (Figure 3C; f, h).

### SLMAP and ER-Golgi transport

**Figure 4:**
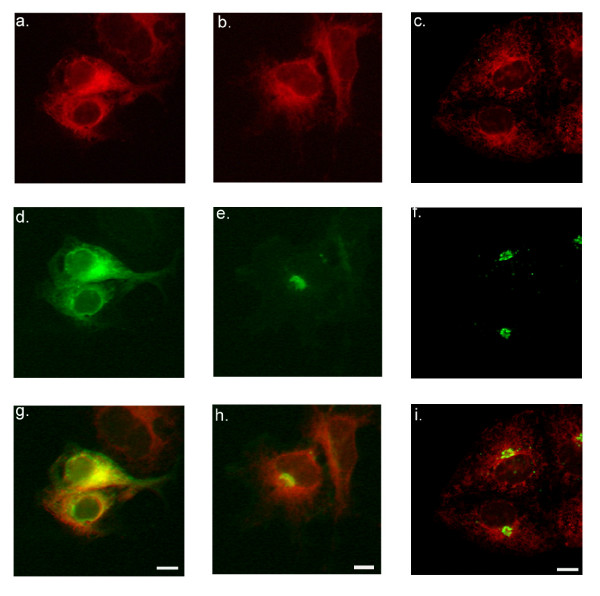
**Overexpression of SLMAP does not affect VSV-G transport from the ER**. COS7 cells were co-transfected with 6Myc-SLMAP and ts045-VSV-G-GFP. When maintained at 40°C (a, d, g), the GFP-tagged viral glycoprotein was retained in the ER (d) and co-distributed with the Myc-labelled SLMAP protein (a). At 32°C, ts045-VSV-G-GFP exited the ER and was observed at perinuclear structures as well as at the cell periphery (e); whereas the 6Myc-SLMAP (b) appeared to remain in the ER. In cells incubated at reduced temperatures (15°C), the GFP-tagged viral glycoprotein is redistributed to perinuclear punctuate-like structures indicative of the ERGIC (f). The localization of the 6Myc-SLMAP (c) remained unaltered in these cells. Overlay of the anti-Myc and GFP signals is shown in g, h, and i.

Comparisons of the predicted primary structure of SLMAP indicates general similarities with coiled-coil TA anchored proteins involved in transport of vesicles from the ER and the docking of vesicles at the Golgi [43,44]. Analysis of the amino acid sequences encoding SLMAPs (Accession No. AAA65597) also indicated the presence of a putative di-acidic sorting signal upstream of the carboxyl-terminal transmembrane domain. (EQE) Di-acidic motifs, defined by Asp/Glu residues separated by a variable residue (D/E)X(D/E), are often located in close proximity to tyrosine-based sorting motifs (YXXö) and are thought to mediate efficient export of the protein from the ER by interacting with the vesicular transport machinery [45,46]. In SLMAP a tyrosine-based sorting motif (YEKT) is present upstream of the di-acidic sorting motif. In view of these features we investigated whether SLMAPs are involved in vesicular transport from the ER to the Golgi. Previous studies have shown that overexpression of coiled-coil membrane proteins involved in vesicle trafficking from the ER to Golgi inhibit protein export from the ER [47]. To examine this possibility, COS7 cells were co-transfected with an ER-localized 6Myc-SLMAP1 and the GFP-tagged glycoprotein of the temperature sensitive strain of vesicular stomatitis virus (ts045-VSV-G-GFP), which serves as a marker of ER to Golgi transport. When maintained at the restrictive temperature (39.5°C), the viral glycoprotein could not be exported from the ER due to a thermoreversible-folding defect (Figure 4d) and consequently co-distributed with 6Myc-SLMAP1 (Figure 4a &4g). Proper folding and subsequent transport out of the ER to the Golgi and cell periphery was achieved when the ts045-VSV-G transfected cells were incubated at the permissive temperature (32°C). Whereas the GFP-labelled viral glycoprotein was transported out of the ER to the Golgi and plasma membrane under conditions that promote vesicular transport (Figure 4e), the 6Myc-SLMAP fusion protein did not appear to exit the ER (Figure 4b) as clearly indicated in the overlay (Figure 4h). Expression of the SLMAP1 lacking either the transmembrane domain or the leucine zipper motifs did not affect the ability of the viral glycoprotein to exit the ER at the permissive temperature (data not shown). COS7 cells co-expressing ts045-VSV-G-GFP and 6Myc-SLMAP1 were also incubated at reduced temperatures to monitor whether SLMAP1 cycles from the ER to the ERGIC [48]. At 15°C the membrane associated SLMAP1 was retained in the ER (Figure 4c), whereas the GFP-labelled viral glycoprotein was visualized as vesicular clusters concentrated at perinuclear sites (Figure 4f) consistent with localization at the ERGIC (Figure 4i). Collectively, these studies indicate that SLMAP1 is not exported via the vesicular transport process nor does elevated expression of SLMAP1 affect transport and is consistent with its absence from the stack Golgi fraction.

### Hydrophobic profiles of the TAs in SLMAP

**Figure 5:**
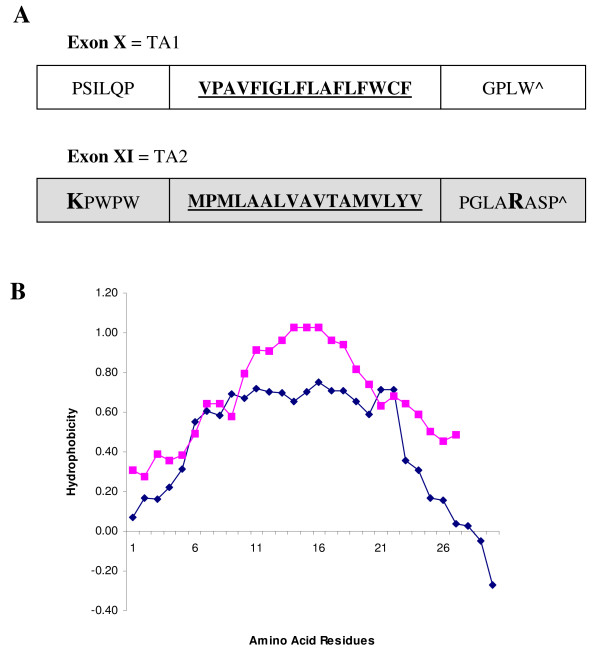
**Primary sequence and hydrophobicity of TAs in SLMAP**. Both of the alternatively spliced SLMAP tail anchors are predicted to encode a transmembrane helix according to the HMMTOP program. The transmembrane regions are shown in bold and underlined, the positively charged residues are shown in a larger font and bold for both TA1 and TA2 (A). The hydrophobicity of the TA1 (pink square) and TA2 (blue diamond) was calculated using the Eisenberg normalised scale, with a window size of 9, the relative weight for window edges was 100 (B).

A single gene encodes SLMAP which undergoes alternative splicing to generate distinct isoforms with unique tail anchors which serve roles in myoblast fusion, centrosomal function and excitation-contraction coupling [29,31,32]. Exon XXIII (of SLMAP) encodes 27 amino acids which comprise the alternatively spliced TA, referred to as TA1. Exon XXIV encodes 30 amino acids which form the constitutively expressed TA, referred to as TA2 [8,29]. To more clearly define SLMAP targeting, the nature of the TAs in SLMAP was explored using the HMMTOP program which predicted that SLMAP's two alternatively spliced C-terminal exons contain a single putative transmembrane domain [49,50] (Figure 5A). TA1, encoded by Exon XXIII, contains a predicted transmembrane helix of 18 amino acids flanked by five amino acid residues at the N-terminal end and four residues at the C-terminal end. TA2, encoded by Exon XXVI, is predicted to contain a transmembrane helix of nineteen amino acids flanked by seven residues at the N-terminal end and five residues at the C-terminus. The two TAs have no sequence homology, but have similar properties, in that they are largely hydrophobic domains (Figure 5B). The major difference between the two TAs is the presence of a basic amino acid residue on either flank of the membrane spanning region in TA2, which are not present in TA1 (Figure 5A).

Numerous reports have shown that the presence of positively charged residues in the regions flanking the membrane spanning region can cause a TA to target the MOM rather that the ER [2,24,25]. A simple examination of the flanking sequences of TA1 and TA2 shows that only TA2 possesses positively charged residues in this region (Figure 5A). This suggests that TA2 may target the MOM whilst TA1 could target the ER. However, some ER targeting TAs possesses positively charged residues in the flanking regions [2,3,14,22], so we might conclude that there may be additional factors involved in the targeting of TAs. It has been shown that the hydrophobicity of the transmembrane region can affect the membrane targeting of a TA [20,27].

The hydrophobicity of the TA1 and TA2 was calculated using the Eisenberg normalised scale with a window size of 9, the relative weight for window edges was 100% (Figure 5B) [51]. The hydrophobicity scores for the two putative trans-membrane domains differ in several regions. TA1 is more hydrophobic over the first five residues which form the N-terminal flanking region and the last five residues, which form the C-terminal flanking region (Figure 5B). However, the largest difference between the SLMAP TAs can be seen in the transmembrane region. TA1 is much more hydrophobic than TA2 between residues 10 and 20, with TA1 having a maximum score of 1.03 whilst TA2 reaches 0.8. This data led us to believe that the difference in hydrophobicity between the two TAs may be enough to result in differential targeting.

### TA2 sequences can target SLMAP1 to mitochondria

**Figure 6:**
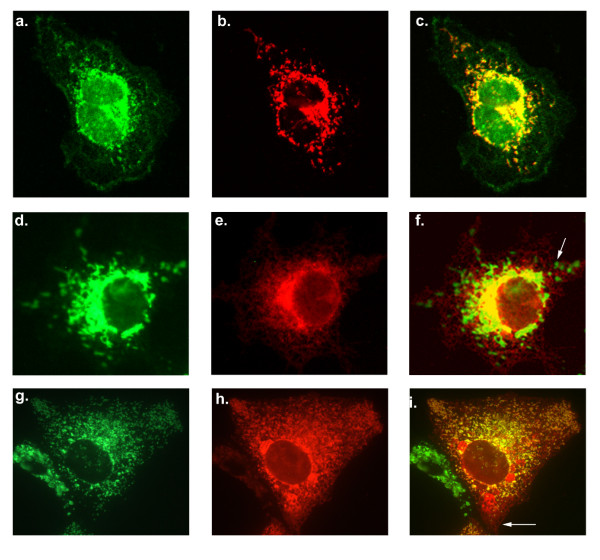
**Endogenous SLMAP localises at the mitochondria and is targeted by the TA2**. Cells were stained with anti-SLMAP (a) and anti-TOM20 (b) to analyze endogenous co-distribution (c). Cos7 cells were transfected with 6Myc-SLMAP1-TA1, and co-stained with anti-Tom20 (d) and anti-6Myc (e), the images were then merged (f) arrow points to an anti-Tom20 stained mitochondria. Cos7 cells transfected with 6Myc-SLMAP1-TA2 and co-stained with anti-Tom20 (g) and anti-6Myc (h) and the images were then merged (i) arrow points to anti-6Myc stained structure with no mitochondria present.

The endogenous localisation of SLMAP in COS7 cells was examined using anti-SLMAP antibodies (Figure 6a) and compared with the mitochondrial marker TOM20 [52] (Figure 6b). The co-distribution (Figure 6c) shows that a significant amount of the endogenous SLMAP co-distributes with TOM20 however there are regions in the cell, where SLMAP is present without TOM-20. These regions would be consistent with the presence of SLMAP at the ER as noted by anti-calnexin staining (Figure 3A; a–c). The subcellular localization of the ectopically expressed SLMAP1 variants encoding TA1 and TA2 were further analysed with respect to Tom20 and MOM localization. Dual immunostaining using anti-Tom20 and anti-Myc antibodies showed that the 6Myc-SLMAP1-TA1 variant did not localize at the mitochondria (Figure 6d–e). A mitochondrion is indicated by the arrow in Figure 6e. Whereas, co-distribution of Tom20 with the 6Myc-SLMAP1-TA2 indicated that this variant may target the MOM, whilst still being present in a filamentous structure throughout the cytoplasm in a similar way to the TA1 construct (Figure 6g–i). The filamentous structure targeted by 6Myc-SLMAP1-TA2 is indicated by an arrow in Figure 6i. It seems clear that SLMAP1-TA2 targets the mitochondria but in addition it also shows co-localization with calnexin at the ER (Figure 2A; d–f) which is distinct from the exclusive localization of SLMAP1-TA1 at the ER (Figure 2A; a–c).

**Figure 7:**
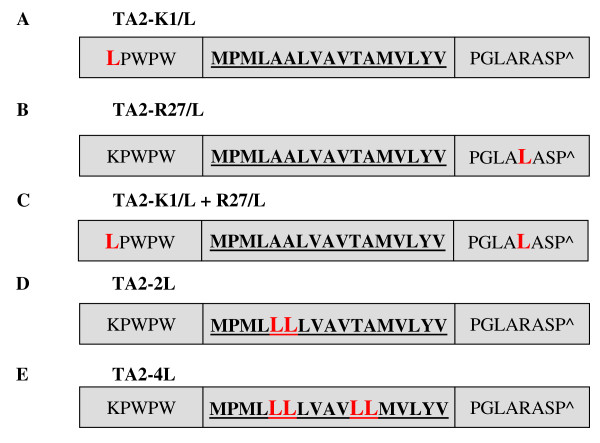
**Primary sequence and membrane helix prediction of the TA2 mutants**. Amino acid substitutions are indicated by red bold letters in a larger case. The amino acid residues are shown using the single letter code and the transmembrane region in each tail anchor is shown in bold and underlined. The sequences shown are from *Mus musculus*.

**Figure 8:**
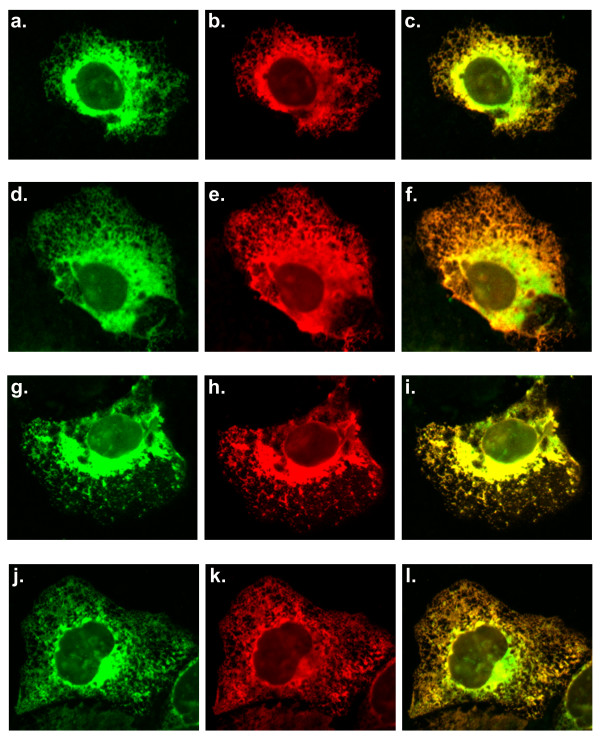
**Targeting of SLMAP1-TA2 and charge**. Wild type GFP-tagged SLMAP-TA2 was co-transfected into Cos7 cells with wild type Myc-tagged SLMAP-TA2 and co-stained with anti-GFP (a) and anti-6Myc antibodies (b) the overlay of the two shows that there is nearly complete overlap with a stronger GFP signal near the nucleus (c). Wild type GFP-tagged SLMAP-TA2 was co-transfected with Myc-tagged SLMAP1-TA2 K_1_/L and co-stained with anti-GFP (d) anti-6Myc (e) the overlay is essentially the same as the control (f). Wild type GFP-tagged SLMAP-TA2 was co-transfected with Myc-tagged SLMAP1-TA2 R_27_/L and co-stained with anti-GFP (g) and anti-6Myc antibodies (h) the overlay is essentially the same as the control (i). Wild type GFP-tagged SLMAP-TA2 was co-transfected with Myc-tagged SLMAP-TA2 K_1_/L + R_27_/L and co-stained with anti-GFP (j) and anti-6Myc (k) antibodies the overlay is essentially the same as the control (l).

In order to determine the molecular properties of TA2 which are responsible for mitochondrial membrane targeting we performed mutational analysis of the tail anchor and flanking regions. We used site-directed mutagenesis to substitute hydrophobic residues for the positively charged residues in the flanking regions to see if TA2 was targeted to the mitochondria via these positively charged residues. The first amino acid in TA2, a lysine residue, was substituted with a leucine residue to remove the N-terminal flanking positive charge, to create the mutant TA2-K_1_/L (Figure 7A). The 27^th ^amino acid, an arginine residue, was substituted with a leucine residue to remove the C-terminal flanking positive charge in the mutant TA2-R_27_/L (Figure 7B). These two mutations were combined in the mutant TA2-K_1_/L+R_27_/L to remove both flanking positively charged residues (Figure 7C). The Myc tagged mutant constructs, SLMAP1-TA2-K_1_/L, SLMAP1-TA2-R_27_/L and SLMAP1-TA2-K_1_/L+R_27_/L were transfected into Cos7 cells and compared with GFP tagged SLMAP1-TA2 for targeting properties. No change in the targeting of SLMAP1-TA2 was observed due to these mutations suggesting that the positive residues are not responsible for the targeting of TA2 to the mitochondria (Figure 8).

**Figure 9:**
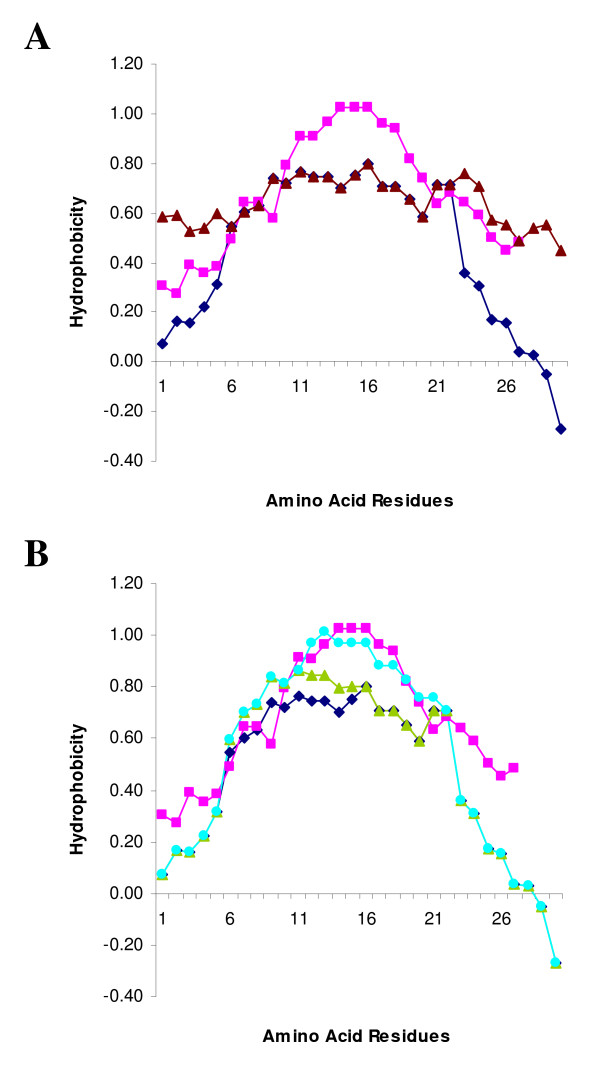
**Hydrophobicity of TA2 mutants**. The hydrophobicity of the TA2-4L (light blue circle) and TA2-2L (green triangle) mutants was calculated using the Eisenberg normalised scale, with a window size of 9, the relative weight for window edges was 100, compared with wild type TA1 (pink square) and TA2 (blue diamond) (A). The hydrophobicity of the TA2-K_1_/L+R_27_/L mutant (brown triangle) was calculated using the Eisenberg normalised scale, with a window size of 9, the relative weight for window edges was 100, compared with wild type TA1 (pink square) and TA2 (blue diamond) (B).

**Figure 10:**
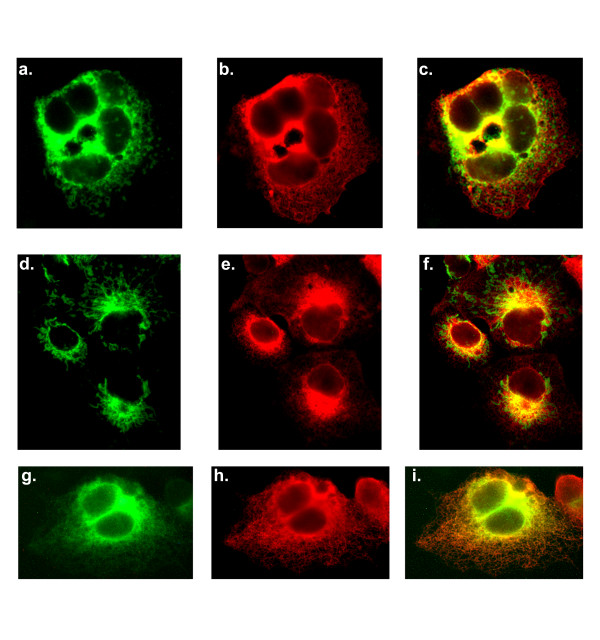
**Targeting of the SLMAP-TA2-4L mutant**. The targeting of SLAMP1-TA2 in Cos7 cells can be altered to that of SLMAP1-TA1 by increasing the hydrophobic moment of the trans-membrane helix with 4 substitutions: A_10_/L, A_11_/L, T_16_/L, and A_17_/L. Wild type GFP-tagged SLMAP1-TA2 was co-transfected with Myc-tagged SLMAP1 TA2 with the 4 substitutions (Myc-TA2-4L) and co-stained with anti-GFP (a) and anti-6Myc antibodies (b) the overlay of the two shows that there is incomplete overlap (c). In Cos7 cells stained with antibodies to TOM20 (d) and transfected with Myc-TA2-4L and stained with anti-6Myc antibodies (e) there are large numbers of mitochondria which do not colocalise with any of the mutant SLMAP (f). Wild type GFP-tagged SLMAP1-TA1 was co-transfected with Myc-TA2-4L and co-stained with anti-GFP (g) and anti-6Myc antibodies (h) when the two images are overlaid there is almost total co-localization (i).

The mutations introduced in TA2 above increased the hydrophobicity of the flanking sequences, without affecting the hydrophobic profile of the transmembrane region itself (Figure 9A). To test whether the hydrophobic properties of the transmembrane sequence in TA2 were important for the targeting of SLMAP1 to the mitochondria we constructed mutants with increased hydrophobicity in their transmembrane domain. Four mutations were made in TA2 to construct a mutant TA2 transmembrane region with similar hydrophobic properties to TA1 (Figure 9B). The alanine residues 10, 11 and 17 along with threonine 16 were all substituted with leucine residues to make SLMAP1-TA2 A_10_/L+A_11_/L+T_16_/L+A_17_/L (SLMAP1-TA2-4L) (Figure 7E). We transfected Myc tagged SLMAP1-TA2-4L into Cos7 cells and co-transfected with wild type GFP tagged SLMAP1-TA2. The TA2-4L mutant did not overlap with the wild type TA2 in many areas in the cell (Figure 10a–c). When we transfected Cos7 cells with the Myc tagged SLMAP1-TA2-4L mutant and co-stained with anti-TOM20 antibodies we saw no overlap with the mitochondrial marker (Figure 10d–f). We co-transfected the Myc tagged SLMAP1-TA2-4L mutant with a GFP tagged wild type SLMAP1-TA1 the observed overlap indicated that both constructs target SLMAP1 to the same cellular compartment (Figure 10g–i). Thus it appears that the TA2-4L mutant shares the targeting properties of wild type TA1.

**Figure 11:**
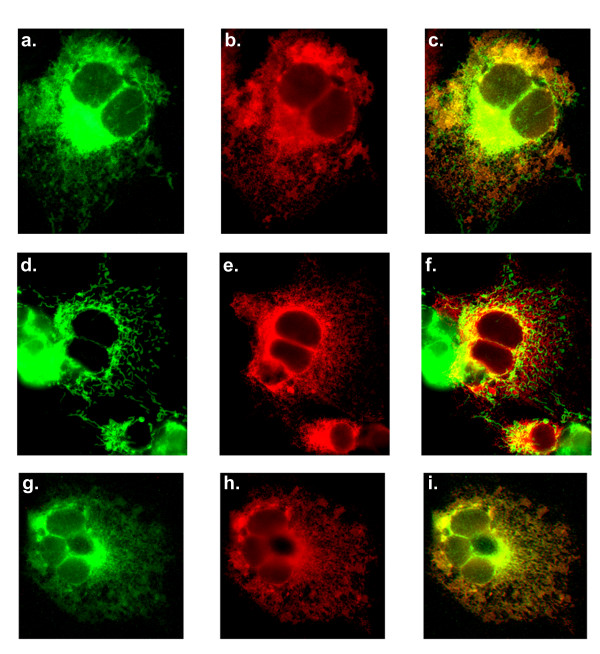
**Targeting of the SLMAP1-TA2-2L mutant**. The targeting of SLAMP1-TA2 in Cos7 cells can be altered to that of SLMAP1-TA1 by increasing the hydrophobic moment of the membrane spanning section with 2 substitutions: A_10_/L and A_11_/L. Wild type GFP-tagged SLMAP1-TA2 was co-transfected with Myc-tagged SLMAP1-TA2 with the 2 substitutions (Myc-TA2-2L) and co-stained with anti-GFP (a) and anti-6Myc antibodies (b) the overlay of the two shows that there is incomplete overlap (c). In cells stained with antibodies to TOM20 (d) and transfected with Myc-TA2-2L and stained with anti-6Myc antibodies (e) there are large numbers of mitochondria which do not co-localize with any of the mutant (f). Wild type GFP-tagged SLMAP1-TA1 was co-transfected with Myc-TA2-2L and co-stained with anti-GFP (h) and anti-6Myc antibodies (i) when the two images were merged there was almost total co-localization (j).

To determine whether a smaller change in hydrophobicity could produce a similar alteration of targeting, we constructed a mutant with two changes in the trans-membrane domain (Figure 9B). The alanine amino acid residues at positions 10 and 11 were substituted with leucine to create SLMAP1-TA2 A_10_/L+A_11_/L (TA2-2L) (Figure 7D). When we co-transfected Myc tagged SLMAP1-TA2-2L with wild type GFP tagged SLMAP1-TA2 in Cos7 cells we observed that the TA2-2L mutant only partially co-localised with TA2 (Figure 11a–c). The Myc tagged SLMAP1-TA2-2L was transfected into Cos7 cells and co-stained with anti-Tom20 antibodies. There was no overlap between the TA2-2L mutant and the MOM marker Tom20 (Figure 11d–f). We co-transfected Cos7 cells with Myc tagged SLMAP1-TA2-2L and wild type GFP tagged SLMAP1-TA1 and observed nearly complete overlap of the two (Figure 11g–i). The results of the TA2-4L and TA2-2L were largely identical, indicating both mutants share the same targeting properties, which were observed to be the same as wild type TA1.

The TA2-4L mutant has a transmembrane region that is only slightly less hydrophobic than the wild type TA1 (Figure 9B) They were found to share similar targeting properties despite the TA2-4L mutant still possessing positively charged amino acid residues in both of the regions flanking its trans-membrane domain, with the corresponding decrease in the hydrophobicity of these regions (compared with TA1) (Figure 7E). The TA2-2L mutant has a much less hydrophobic trans-membrane region than both TA1 and TA2-4L, but is still more hydrophobic than wild type TA2 over amino acids 6–15 (Figure 9B). However, it also shares the same targeting properties as TA1; despite it like TA2-4L possessing flanking positively charged amino acids (Figure 7D). It appears from this data that the TA2 transmembrane region is at the very edge of the hydrophobic range which will target a TA to the MOM. This may be the reason that TA2 is promiscuous in targeting SLMAP1 to mitochondria and ER.

The TA2-K_1_/L+R_27_/L mutant which has the positively charged amino acids replaced with leucine does have an increase in hydrophobicity compared with wild type TA2 but only in the regions flanking the transmembrane domain (Figure 9A). The hydrophobicity of the trans-membrane region of the TA2-K_1_/L+R_27_/L mutant remains the same as the wild type TA2 trans-membrane region (Figure 9A). These data suggest that the targeting of TA2 to the mitochondria is due to the reduced hydrophobicity of the transmembrane region in comparison with the TA1 transmembrane region rather than the presence of the positively charged amino acid residues in the flanking sequences.

## Discussion

Tail anchored (TA) membrane proteins are important in several cellular processes including neurotransmitter release, vesicle transport, membrane fusion and signal transduction [1-8]. All tail anchors comprise a C-terminal hydrophobic transmembrane region which may be flanked by positively charged residues which are promiscuous in terms of sub-cellular targeting [10]. These features are present in the SLMAP C-terminal region and may account for its distinct pattern of localization observed here in the liver versus Cos 7 cells compared with the cardiomyocytes [31]. It is notable that endogenous SLMAP co-localised with the ER marker calnexin and the mitochondrial marker TOM20 implying that it is like other TA proteins such as BCL2 which also reside in these distinct membranes [5]. The different TAs utilized by SLMAP appear to be responsible for the targeting of SLMAP1 to distinct subcellular structures including the ER and mitochondria, perhaps the MOM.

The information required for the targeting of SLMAP to intracellular membranes was not contained in specific amino acids that flank the predicted transmembrane domain but was dictated by the overall hydrophobic profile of the TA. We found that SLMAP1 could target either the ER alone or the ER and the mitochondria in Cos7 cells depending on which of the two alternative tail anchors (TA1 or TA2) were being expressed. The disruption of the cytoskeleton with nocodazole showed that the distribution patterns of SLMAP1 are dependent on an intact microtubule network. This is wholly consistent with changes in distribution noted for other ER resident proteins, and the known role of the cytoskeleton in anchoring intracellular membranes in place [39,53]. It is interesting to speculate that SLMAP may act as a potential molecular link between intracellular membranes and the microtubule based cytoskeleton. Several membrane associated proteins provide a link between intracellular membranes and the microtubule cytoskeleton including CLIPs [53] and the ER membrane protein p63 [39,54]. Such interactions, which may also involve SLMAP, are crucial for the positioning and structural maintenance of subcellular organelles [55].

Tail anchors comprise a single membrane spanning helix of 17 to 21 amino acid residues at the C-terminal end of the protein [9,10]. Investigations have shown that the replacement of basic residues in the regions flanking the membrane helix with hydrophobic residues can alter the targeting of TAs [2,24,25]. It would appear that the presence of positively charged residues in the flanking regions increases the likelihood of the TA targeting the MOM instead of the ER [26].

The two different tail anchors in SLMAP are generated by alternative splicing and both appear to target the protein to subcellular locations. Both TA1 and TA2 target SLMAP1 to the ER. TA2 also targets SLMAP1 the mitochondria as evidenced by co-localization with Tom20. We found that this differential targeting was due to the lower hydrophobic content of the trans-membrane helix in TA2 when compared to the alternatively spliced TA1. Immunohistochemistry indicates that TA1 directs SLMAP1 predominantly to the ER and not the mitochondria probably due to the more hydrophobic nature of the transmembrane domain. We were able to prevent TA2 from targeting the mitochondria by the substitution of moderately hydrophobic amino acids with the highly hydrophobic leucine residues implying that the important targeting information in TA2 is the relative hydrophobicity of the transmembrane region. In this regard Bielharz *et al*., [20] observed a similar shift in the targeting of Fis1 from the MOM to the ER when the hydrophobicity of its TA was increased.

We found no evidence to suggest that the positively charged amino acids which flank the membrane spanning helix are responsible for the targeting of TA2 to the mitochondria per-se. This is in contrast to the view that basic amino acids flanking the TA are responsible for MOM targeting [2,7,14,24,25]. It seems more likely that in these cases the substitution of the positively charged, and therefore highly hydrophilic, amino acids results in an increase in the overall hydrophobicity of the TA and altered targeting. However, this did not occur in our TA2 K_1_/L R_27_/L mutants despite the hydrophobicity of the flanking regions being increased to a level above that of the TA1 isoform. Recently it has been shown that artificial TAs require a moderately hydrophobic transmembrane region to target the MOM [28]. Consistent with this speculation, the directed substitution of 2 amino acid residues in the flanking region in TA2 of SLMAP1 did not sufficiently change hydrophobic nature to shift targeting.

The presence of the two alternatively spliced exons encoding tail anchors which target SLMAP1 to different locations suggests SLMAP1 may perform multiple subcellular functions. Given the coiled-coil nature of the majority of the SLMAP protein, it may be that it tethers proteins into place on both the ER and perhaps the MOM. Alternatively, it may be involved in tethering of the two membrane systems together through homo/hetero-dimerisation. Differential levels of expression of SLMAP with the different TAs, suggest that SLMAP may serve varying subcellular roles in tissue specific manner [8,30,31]. In this regard, the expression levels of SLMAP were found to be important for normal myoblast fusion [29]. SLMAP was not found to be localized with the vesicle transport mechanism nor did its over expression affect vesicle transport in Cos7 cells, this implies that it is not involved in intracellular trafficking.

While the mechanism of TA protein membrane insertion remains to be defined, it appears that both the SRP (signal recognition particle) and a novel mechanism utilizing ATP appear to be involved [56,57]. Which of these mechanisms is utilised by SLMAP remains unknown.

## Conclusion

Our data here supports the view that the sub-cellular targeting of SLMAP is determined by the overall hydrophobicity of its alternative tail-anchor (TA). Further, the basic amino acid residues in the regions flanking the transmembrane helix contribute to the overall hydrophobic profile of the TA and its targeting rather then the individual residues being a targeting signal. Further more, the potential association of SLMAP with the MOM implies additional roles for this molecule in mitochondrial function.

## Methods

**Membrane helix predictions **were performed using the HMMTOP program [49,50]. **Hydropathy calculations **were performed using the ProtScale tool  with the Eisenberg algorithm using a window size of 9 and the relative weight for the window edges was 100%. [51].

### DNA constructs

Since the targeting of the tail anchored proteins resides at the C-terminal regions in general we used GFP and 6Myc-tagged expression constructs of the naturally occurring SLMAP1-TA1, SLMAP1-TA2, as previously described [29,31,32]. **Site directed mutagenesis **was performed using the Stratagene "Quikchange" kit. Template DNA concentration was 30 μg per reaction. The K_1_/I mutant (AAA/A**T**A) was created using 5' (GGA AAT AAT A**T**A CCC TGG CCC) and 3' (GGG CCA GGG T**A**T ATT ATT TCC). The R_27_/I mutant (AGA/A**T**A) was created using 5' (CCA GGT CTG GCC A**T**A GCT TCT CCG TG) and 3' (CA CGG AGA AGC T**A**T GGC CAG ACC TGG). The K_1_/I+R_27_/I mutant was created by performing SDM on the K_1_/I mutant using the R_27_/I primers (5' (CCA GGT CTG GCC A**T**A GCT TCT CCG TG) and 3' (CA CGG AGA AGC T**A**T GGC CAG ACC TGG)). The P_4_/L mutant (CCC/CTC) was created using 5' (ATA CCC TGG C**T**C TGG ATG CCC) and 3' (GGG CAT CCA GAG CCA GGG TAT). The A_10_/L + A_11_/L mutant (GCT/CTT + GCC/CTC) was created using 5' (ATG CCC ATG TTG **CT**T **CT**C CTG GTT GCG GTG) and 3' (CAC CGC AAC CAG G**AG **A**AG **CAA CAT GGG CAT). The T_16_/L + A_17_/L mutant (ACA/CTA + GCT/CTC) was created using 5' (CTG GTT GCG GTG **CT**A **CTC **ATC GTG CTG TAT) and 3' (ATA CAG CAC GAT **GAG **T**AG **CAC CGC AAC CAG). The A_10_/L+A_11_/L+T_16_/L+ A_17_/L mutant was created by SDM on the A_10_/L+A_11_/L construct using the T_16_/L + A_17_/L creation primers (5' (CTG GTT GCG GTG **CT**A **CTC **ATC GTG CTG TAT) and 3' (ATA CAG CAC GAT **GAG **T**AG **CAC CGC AAC CAG)).

### Cell culture, transfections and cytoskeletal disruptions

COS7 African green monkey kidney and C2C12 cells were maintained at 37°C in Dulbecco modified essential media (DMEM) supplemented with 10% heat inactivated fetal bovine serum and antibiotics. Transient transfection experiments were performed using the fugene™ (Roche Biochemicals) transfection reagent according to the manufacturers' specifications. Disruptions of microtubules were induced in COS7 cells with 10 μM nocodazole (Sigma-Aldrich) for two hours at 37°C. Filamentous actin was disrupted in COS7 cells with 1 μM cytochalain D (Sigma-Aldrich) for 2 hours at 37°C. **Cell Culture and Immunohistochemistry**. Transfections were performed as previously described [30], and visualised using specific antibodies, anti-Myc 9E10 (Roche), anti-TOM20 (Santa Cruz), anti-GFP (Roche), and anti-Calnexin (StressGen) with an Axiophot (Carl Zeiss) fluorescent microscope and images captured as described previously [29]. For endogenous co-localisation studies either C2C12 cells or COS7 cells were stained with anti-SLMAP antibodies and either anti-Calnexin or anti-Tom20.

### Subcellular fractionation of the Golgi and ER

Stacked Golgi fractions (SGF) and endoplasmic reticulum fractions were isolated from rabbit liver according to the method described by Taylor *et al*., [34]. All procedures were performed on ice in the presence of protease inhibitors (PMSF, leupeptin, aprotonin, pepstatin A). Freshly removed livers were minced and then homogenized (0.5 M phosphate buffered sucrose. 100 mM KH_2_PO_4_/K_2_HPO_4 _pH 6.5 and 5 mM MgCl_2_). The homogenate was centrifuged at 1500 × g for 10 minutes to remove unbroken cells, debris and nuclei. Postnuclear supernatant (PNS) was loaded onto a sucrose step gradient (1.3 M sucrose, 0.86 M sucrose, PNS in 0.5 M sucrose, 0.25 M sucrose) and centrifuged at 100,000 × g for one hour. Fractions collected from the gradient included S1 (0.25–0.5 M interface), A1 (0.5 M layer), S2 (0.5–0.86 M interface), B1 (0.86 M layer), S3 (0.86–1.3 M interface), C1 (1.3 M layer), and pellet (P). S2 fraction was adjusted to 1.15 M sucrose, and then overlaid with 1.0 M sucrose, 0.86 M sucrose and 0.25 M sucrose. This sucrose gradient was centrifuged at 76,000 × g for 3 hours. Fractions collected included: SGF1 (0.26–0.86 M interface), SGF2 (0.86–1.0 M interface), C2 (1.0 M layer), SGF3 (1.0–1.15 M interface), and SGFL (1.15 M layer). Protein content of each fraction was determined via the BCA protein assay (Pierce). Equal amounts (10 μg) of each fraction were loaded onto a 10% SDS polyacrylamide gel. **ER-Golgi protein transport assays **The ts045-VSVG-GFP expression plasmid was donated by Dr. Lippincott-Schwartz [37]. The plasmid was co-transfected with 6Myc-tagged SLMAP-pcDNA3 constructs into COS7 at 40°C for 15 hours. Cells were then shifted to either 32°C for one hour or 15°C for three hours to monitor the transport of ts045-VSVG-GFP from the ER to the ERGIC, Golgi and plasma membrane.

## Authors' contributions

JB conducted the experiments on the site directed mutagenesis and the hydrophobicity predictions while RG carried out the trafficking and biochemical studies and MS analyzed sequences and made constructs. BST conceptualized the experiments and drafted the manuscript together with JB, RG and MS. All authors read and approved the final manuscript. JTB and RMG contributed equally to this work.
